# Multidimensional Regulatory Mechanisms of *LvChia2* on Growth in the Pacific White Shrimp (*Litopenaeus vannamei*)

**DOI:** 10.3390/genes16091110

**Published:** 2025-09-19

**Authors:** Shangyi Li, Yifan Lei, Qingyun Liu, Qiangyong Li, Chunling Yang, Yuliu Huang, Digang Zeng, Liping Zhou, Min Peng, Xiuli Chen, Fan Wang, Yongzhen Zhao

**Affiliations:** 1 Department of Biology, College of Science, Shantou University, Shantou 515063, China; uk_lsy@163.com (S.L.); leiyf_2000@163.com (Y.L.); 20lpzhou@stu.edu.cn (L.Z.); 2 Guangxi Key Laboratory of Aquatic Genetic Breeding and Healthy Aquaculture, Guangxi Academy of Fishery Sciences, Nanning 530021, China; liuqy198704@163.com (Q.L.); lqy_cz@163.com (Q.L.); scsycl@163.com (C.Y.); 15878757632@163.com (Y.H.); zengdigang@126.com (D.Z.); gxnnpm@126.com (M.P.); chenxiuli2001@163.com (X.C.)

**Keywords:** *LvChia2*, *Litopenaeus vannamei*, molecular breeding, growth regulatory network

## Abstract

**Background**: As a globally significant aquaculture species, elucidating the molecular mechanisms underlying the regulation of the Pacific White Shrimp (*Litopenaeus vannamei*) growth holds substantial scientific and industrial value. This study systematically investigates the role of the *LvChia2* gene in governing growth and development through a cross-tissue metabolic network approach. **Methods**: RNA knockdown (RNAi)-mediated knockdown of *LvChia2* significantly impaired growth performance and triggered a tissue-specific metabolic compensation mechanism. **Results**: This mechanism was characterized by reduced crude lipid content in muscle and adaptive modulation of lipase (LPS) activities in hepatopancreatic and intestinal tissues, suggesting inter-tissue metabolic coordination. Transcriptomic profiling identified 610 differentially expressed genes (DEGs), forming a three-dimensional regulatory network encompassing “energy metabolism, molt regulation, and nutrient utilization.” Key mechanistic insights revealed the following: (1) Enhanced mitochondrial energy transduction through the upregulation of ATP synthase subunits and NADH dehydrogenase (*ND-SGDH*). (2) The disruption of ecdysteroid signaling pathways via suppression of Krueppel homolog 1 (*Kr-h1*). (3) The coordinated regulation of nitrogen metabolism through the downregulation of glutamine synthetase and secretory phospholipase A2. These molecular adaptations, coupled with tissue-specific oxidative stress responses, reflect an integrated physiological strategy for environmental adaptation. **Conclusions**: Notably, this study provides the first evidence in crustaceans of chitinase-mediated growth regulation through cross-tissue metabolic interactions and identifies six core functional genes (*ATP5L*, *ATP5G*, *ND-SGDH*, *Kr-h1*, *GS*, *sPLA2*) as potential targets for molecular breeding. A novel “gut-hepatopancreas axis” energy compensation mechanism is proposed, offering insights into resource allocation during metabolic stress. These findings advance our understanding of crustacean growth regulation and establish a theoretical foundation for precision aquaculture strategies, including genome editing and multi-trait genomic selection.

## 1. Introduction

The Pacific White Shrimp (*L*. *vannamei*) is the most commercially valuable species in global aquaculture, accounting for over 80% of total farmed shrimp production and representing an industry valued at over $10 billion [1,2]. This species has dominated global markets since large-scale farming commenced in the 1970s, due to its biological advantages, including euryhalinity (5–40‰ salinity tolerance), high-density tolerance (60–150 individuals/m^2^), and a short growth cycle (reaching market size within 100 days) [3]. In recent decades, significant efforts have been made to enhance its economic traits, such as growth rate, disease resistance, and tolerance to extreme temperature and salinity conditions [4,5,6]. Among these traits, growth rate is one of the most critical economic factors in aquaculture, closely linked to profitability, which makes its enhancement a primary focus of shrimp breeding programs.

To date, omics technologies including Quantitative Trait Locus (QTL) mapping and Genome-Wide Association Study (GWAS) have identified numerous candidate genes associated with growth traits in *L. vannamei* [7], such as *AMY2*, *CTSL* [8], *STEAP4* [9], and *BAMBI* [10]. These findings reveal the complex regulatory mechanisms underlying muscle development and energy metabolism. However, growth traits result from intricate genetic networks influenced by multiple factors, and research on growth-related genes and their regulation in crustaceans remains limited.

The unique intermittent growth pattern of crustaceans, which relies on periodic molting for somatic expansion, is closely linked to the dynamic balance of chitin metabolic pathways [11]. Chitinases, the key enzymes responsible for chitin degradation, exhibit significant functional diversity across different species [12]. While research on insect chitinases has achieved considerable depth, encompassing gene cloning, molting cycle dynamics, RNA knockdown (RNAi) functional analyses [13], endogenous hormonal regulation, environmental pollutant impacts, heterologous expression, and disease control [14,15], studies on chitinases in aquatic crustaceans remain in their infancy and lack systematic frameworks.

In crustaceans, chitinases, which are members of glycoside hydrolase family 18 (GH18), not only degrade the old exoskeleton but also indirectly enhance growth performance by modulating gut microbiota homeostasis and immune defenses [16]. Notably, Group 2 chitinases within the GH18 family exhibit tissue-specific expression dynamics during molting cycles. For instance, in the mud crab (*Scylla paramamosain*), the expression of *SpChia2* is relatively low during the D1 stage of the molting cycle but declines sharply after the D2 stage (pre-molt phase) [17]. Similarly, expression profiling of *PmChia2* in the black tiger shrimp (*Penaeus monodon*) revealed peak expression during the pre-molt phase [18]. These stage-specific expression patterns align with the high demand for chitin degradation in the old exoskeleton, suggesting that Group 2 chitinases spatiotemporally regulate the enzymatic hydrolysis of the old cuticle to facilitate subsequent epidermal remodeling. Remarkably, RNAi-mediated silencing of *LsChia2* in the salmon louse (*Lepeophtheirus salmonis*) significantly suppresses gene expression during the larval stage, leading to axial developmental defects, impaired locomotion, and reduced host infectivity [19,20]. Collectively, these findings highlight the evolutionarily conserved role of Group 2 chitinases as core effectors in crustacean molting, driving the programmed degradation of the old exoskeleton. However, their potential for genetic improvement of growth traits in *L. vannamei* remains underexplored.

Transcriptome sequencing analysis, a widely utilized method for studying non-model organisms, facilitates a systematic investigation of gene expression under specific conditions and the elucidation of regulatory networks [21]. In this study, we analyzed previously generated transcriptomic data [22] to screen for and identify the growth-related gene *Chia2* in *L. vannamei* with divergent growth rates. Following the cloning and characterization of its gene structure, phylogenetic relationships, and expression patterns, we employed RNAi [13] to knock down *LvChia2* expression and observed associated growth phenotypes and transcriptional changes. Our results suggest that *LvChia2* may regulate shrimp growth through pathways involving lipid and glucose metabolism. This study provides insights into the molecular mechanisms underlying *LvChia2*-mediated growth regulation in *L. vannamei* and offers valuable genetic information for developing molecular breeding strategies in shrimp aquaculture.

## 2. Materials and Methods

### 2.1. Ethics Statement

All procedures for animal breeding, handling, and sampling in this study received approval from the Animal Care and Ethics Committee of Guangxi Academy of Fishery Sciences (Animal ethics approval number: 2025GAFS09), thereby affirming the trustworthiness and ethical conduct of our research.

### 2.2. Experimental Animals and Aquaculture Management

Specific pathogen-free (SPF) post-larvae of *L. vannamei* were obtained from the Guangxi Academy of Fishery Sciences (Nanning, China). A total of 150 healthy shrimp, with a uniform body weight of 1.22 ± 0.08 g, were randomly distributed into three circular polyethylene tanks (dimensions: 97 × 81 × 73 cm), at a density of 50 shrimp per tank. Prior to start of the experiment, the shrimp underwent a 5 d acclimation time, During this time, they were maintained under carefully controlled environmental conditions: salinity was set at 25‰ (achieved by diluting brine, followed by 24 h sterilization, 24 h dechlorination, and 24 h aeration), water temperature was maintained between 26 and 28 °C, pH levels were regulated within the range of 7.8–8.2, dissolved oxygen (DO) was ensured to be no less than 6 mg/L, ammonia nitrogen concentration was kept below 0.2 mg/L, and nitrite concentration was maintained at less than 0.01 mg/L. The shrimp were fed commercial feed (nutritional composition: 38% crude protein, 4% crude fat, 8% crude fiber, 16% ash and 11% moisture, supplied by Fujian Tianma Technology Group Corporation, Fujian, China) at a rate of 5% of their body weight, administered three times daily (at 10:00, 16:00, and 22:00), with adjustments made based on their feeding response. To maintain water quality, one-third of the water volume in each tank was replaced daily. For the RNAi experiment, after the acclimation phase, shrimp exhibiting uniform size and weight were selected. Their initial body weight and length were measured precisely using an analytical balance (precision: 0.1 mg) and a digital caliper (precision: 0.1 mm), respectively. These measurements were repeated every 5-day throughout a 20-day trial period to monitor growth dynamics.

### 2.3. Sample Collection and Analysis

Following a 24 h starvation period to standardize the physiological state of the shrimp, several growth parameters were calculated, including survival rate (SR), weight gain rate (WGR), condition factor (CF), and specific growth rate (SGR), using the established formulas outlined below:

SR (%) = (Final shrimp count/Initial shrimp count) × 100

WGR (%) = [(Final weight − Initial weight)/Initial weight] × 100

Length gain (%) = [(Final length − Initial length)/Initial length] × 100

CF (100 g/cm^3^) = (Body weight/Body length^3^) × 100

SGR (%/d) = [ln(Final weight) − ln(Initial weight)]/Days × 100

Tissue samples, including muscle, hepatopancreas, and intestinal tissues, were collected from 12 shrimp per group. These samples were immediately flash-frozen in liquid nitrogen and stored at −80 °C for subsequent analyses. Muscle tissues were reserved for transcriptomic sequencing and proximate composition analysis, while hepatopancreas and intestinal tissues were used for enzymatic activity assays.

### 2.4. Transcriptomic Screening of Growth-Related Genes

Muscle tissues were collected from 30 fast-growing and 30 slow-growing individuals of *L. vannamei*, selected based on significant differences in both body weight and length. Total RNA extraction was performed using TRIzol reagent, followed by quality assessment through 1% agarose gel electrophoresis and NanoDrop 2000 spectrophotometry (Thermofisher, MA, USA), ensuring an OD260/280 ratio within the range of 1.8–2.2. Sequencing libraries were constructed utilizing the Illumina TruSeq RNA Library Prep Kit and subjected to paired-end sequencing, generating 150 bp reads on the Illumina HiSeq™ 2500 platform (IIlumina, San Diego, CA, USA). Raw sequencing reads underwent rigorous preprocessing with Trimmomatic to remove adapter sequences, low-quality bases (Phred score < 20), and ambiguous reads. High-quality clean reads were then aligned to the *L. vannamei* reference genome (ASM378908v1) using HISAT2 [23]. Transcript assembly and quantification were conducted via StringTie, and DEG were identified using DESeq2, with thresholds set at |log_2_(fold change)| ≥ 1 and adjusted *p* < 0.05. Functional annotation of DEGs was performed against multiple databases, including Kyoto Encyclopedia of Genes and Genomes (KEGG), Gene Onthology (GO), Pfam, and Swiss-Prot [22].

Nine candidate genes exhibiting consistent upregulation (fold change ≥ 2) in fast-growing shrimp were prioritized for validation. Gene-specific primers, designed using Primer Premier 6.0, were synthesized with amplicon lengths ranging from 150 to 300 bp and annealing temperatures set between 55 and 60 °C. Quantitative PCR (RT-qPCR) assays were performed on 20 muscle tissue samples, with β-actin serving as the reference gene. Linear regression models were employed to establish correlations between gene expression levels and body weight, leading to the identification of *LvChia2* as the most robust candidate gene associated with growth traits.

### 2.5. Molecular Cloning and Characterization of LvChia2

Total RNA extraction from muscle tissues was performed using TRIzol reagent (Takara, Kusatsu, Japan). Briefly, 100 mg of tissue was homogenized in liquid nitrogen, lysed in RNAiso Plus (Takar, Kusatsu, Japan), and treated with chloroform. RNA precipitation was achieved using isopropanol, followed by washing with 75% ethanol and dissolving the RNA in RNase-free water. RNA integrity was confirmed via electrophoresis, and concentration was measured using a NanoDrop 2000 spectrophotometer. cDNA synthesis was conducted using the PrimeScript™ FAST RT Reagent Kit (Takara, Kusatsu, Japan), which included genomic DNA removal with gDNA Eraser (42 °C, 2 min) and reverse transcription (37 °C, 10 min). The *LvChia2* open reading frame (ORF) was amplified using primers designed based on transcriptomic data. PCR conditions were optimized as follows: initial denaturation at 95 °C for 3 min; 35 cycles of denaturation at 95 °C for 30 s, annealing at 55 °C for 30 s, and extension at 72 °C for 2 min; followed by a final extension step at 72 °C for 10 min. PCR products were gel-purified using a DiaSpin DNA Gel Extraction Kit (Sangon Biotech, Shanghai, China), ligated into the pMD19-T vector, and transformed into *Escherichia coli* (DH5α) competent cells. Positive clones were verified through colony PCR and Sanger sequencing.

A comprehensive analysis of the *LvChia2* nucleotide sequence and the deduced amino acid sequence was conducted using ORF Finder (NCBI) and ExPASy Translate. Protein domain prediction was performed using the SMART tool, and phylogenetic trees were constructed with MEGA 12.0, employing the Neighbor-Joining method with 1000 bootstrap replicates (Institute of Molecular Evolutionary Genetics, State College, PA, USA). Alignment maps of conserved amino acid sites were generated using DNAMAN 9.0 software to further characterize the evolutionary conservation and structural features of *LvChia2*.

### 2.6. Developmental Expression Profiling

The expression profile of *LvChia2* was evaluated across six distinct developmental stages of *L. vannamei*: mysis larvae (6 d post-hatching, DPH), post-larvae (12 DPH), juveniles (24 DPH), subadults (50 DPH), pre-adults (72 DPH), and adults (100 DPH). Muscle tissues from three biological replicates at each stage were collected and analyzed to ensure robust and representative data across the entire developmental spectrum of the shrimp.

### 2.7. RNAi and Functional Analysis

Two small knockdown RNA (siRNA) duplexes targeting *LvChia2* (GenBank: LOC113830447) were meticulously designed using the siDirect 2.0 and DSIR algorithms, with rigorous measures implemented to minimize off-target effects, as confirmed by NCBI BLAST analysis (https://blast.ncbi.nlm.nih.gov/Blast.cgi) (accessed on 10 May 2025). These siRNA sequences were chemically synthesized by Sangon Biotech and included dTdT overhangs and phosphorothioate modifications to enhance stability and efficacy. A scrambled siRNA (siNC) was synthesized as a negative control to account for non-specific effects. Eighty-four shrimps were randomly allocated into three groups: the siChia2 group (experimental), the siNC group (negative control), and the DEPC-water group (blank control). The siRNA solution (0.1 μg/mL) was administered via intramuscular injection into the second abdominal segment of the shrimp at a dosage of 5 μg/g of body weight, with injections repeated every 5 days for a total duration of 20 days. Tissue samples, including muscle, hepatopancreas, and intestinal tissues, were collected at multiple time points post-injection (24, 48, 72, 96, and 120 h) for RNA extraction and RT-qPCR validation to assess knockdown efficiency. Additionally, samples were collected 20 days post-injection to verify the subsequent functional impacts.

### 2.8. Quantification of mRNA by RT-qPCR

Total RNA was extracted from the shrimp muscle using TRIzol^®^ Reagent (Ambion, Austin, TX, USA). cDNA was synthesized using the FastKing one-step genome-removing method (TianGen, Beijing, China) and used as the template for RT-qPCR. The RT-qPCR was performed with TB Green^®^ Premix Ex Taq (Takara, Kusatsu, Japan) to quantify mRNA levels. The relative fold change in mRNA expression was calculated by the 2^−ΔΔCt^ method with β-actin serving as an internal control. Each sample was tested in triplicate. The RT-qPCR procedure consisted of one cycle at 95 °C for 30 s, followed by 40 cycles of 95 °C for 5 s and 60 °C for 20 s, concluding with a melting curve analysis from 65 °C to 95 °C. All primers are listed in Table 1.

### 2.9. Proximate Composition and Enzyme Activity Assays

Proximate composition analysis of muscle tissues was conducted to determine the contents of crude protein, lipids, ash, and moisture, following the established Association of Official Analytical Chemists (AOAC) (2005) protocols. Additionally, enzymatic activity assays were performed for lipase (LPS), amylase (AMS), malondialdehyde (MDA), catalase (CAT), and total antioxidant capacity (TEAC), utilizing commercial kits provided by the Nanjing Jiancheng Bioengineering Institute. These analyses aimed to elucidate the physiological and biochemical changes associated with the knockdown of *LvChia2*.

### 2.10. RNA-Seq Analysis

Total RNA was extracted from *LvChia2*-knockdown (siChia2) and control (siNC) shrimp, with only samples exhibiting a knockdown efficiency of ≥70% selected for sequencing. Sequencing was performed on the NovaSeq X Plus and DNBSEQ-T7 platforms to ensure high-throughput and high-quality data generation. Libraries were constructed using the NEBNext Ultra II RNA Library Prep Kit (NEB, Lpswich, MA, USA), which included steps such as mRNA enrichment (via Oligo dT beads), fragmentation (target size: 300 bp), cDNA synthesis, adapter ligation, and PCR amplification. Raw reads were filtered using Trimmomatic to remove low-quality sequences and contaminants. Subsequent alignment to the *L. vannamei* genome (RefSeq: GCF_003789085.1) was conducted using HISAT2 [23]. DEGs were identified using DESeq2, with thresholds set at |log_2_FC| ≥ 1 and *p* < 0.05. Functional annotation of DEGs was performed using the KEGG and GO databases to gain insights into the biological pathways and processes affected by *LvChia2* knockdown. Three significantly upregulated and three downregulated DEGs were selected, and their expression was validated via quantitative real-time PCR (RT-qPCR) to elucidate the role of candidate genes in growth rate.

### 2.11. Statistical Analysis

All data are presented as means ± standard deviations (SD) to provide a clear representation of the variability within the dataset. Statistical significance between groups was determined using a one-way ANOVA, conducted with SPSS version 26.0 software. Significance thresholds were set at *p* < 0.05 (*) and *p* < 0.01 (**), ensuring a rigorous evaluation of the experimental results and providing a solid foundation for the conclusions drawn from the data analysis.

## 3. Results

### 3.1. LvChia2 Sequence and Bioinformatics Analysis

**Figure 1:**
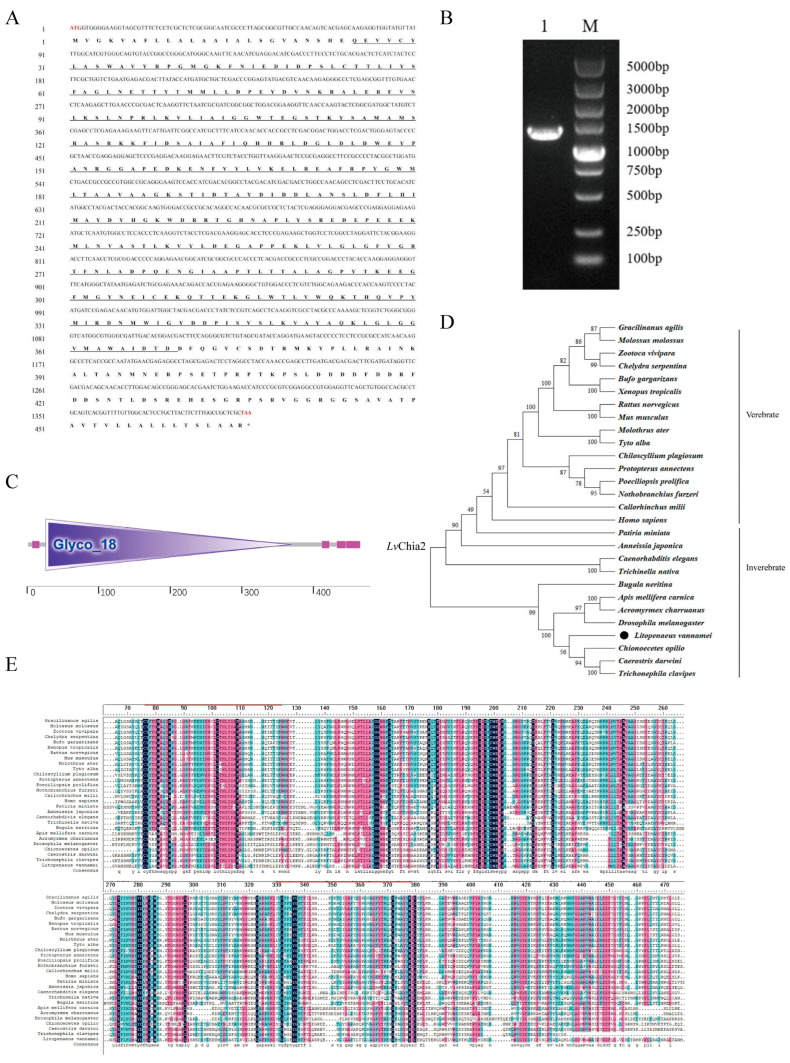
*LvChia2* sequence and bioinformatics analysis. (**A**) The nucleotide sequence and the deduced amino acid sequence of *LvChia2*. The start codon and stop codon are marked in red font, and the underlined parts indicate the domain positions (**B**) Electrophoresis of *LvChia2*. (**C**) The domain architecture of *LvChia2* was predicted by SMART. (**D**) Phylogenetic tree of aligned amino acid sequences of *LvChia2*; the black circle marked *LvChia2*. (**E**) Protein sequence alignment of *LvChia2* protein from shrimp and that from other species.

Based on the cross-family transcriptome comparative analysis and screening of 9 significantly upregulated (Fold Change ≥ 2.0, False Discovery Rate (FDR) < 0.05) candidate genes obtained from the previous research results of the research group, cDNA synthesis and RT-qPCR verification were carried out. The muscle RNA was extracted from 20 individuals of shrimp belonging to the same family and of the same age (66 d) but exhibiting significant variations in body weight ranging from 2 to 25 g for RNA sequencing. Through linear regression analysis, it was determined that the relative expression level of the *LvChia2* gene exhibited the most significant positive correlation with individual body weight, with a coefficient of (R^2^) reaching 0.4903 (Figure S1). This result was notably superior compared to other candidate genes, suggesting that *LvChia2* may be a key functional gene regulating the growth and body weight of shrimp. The full-length cDNA sequence of *LvChia2* was 1401 bp, encoding 466 deduced amino acids (Figure 1A), and was cloned based on transcriptome data and the reference genome (Figure 1B). *LvChia2* was predicted to possess a domain, with its mature form consisting of 344 aa (MW 38kDa) and a pI value of 5.01, which includes a Glycoside hydrolase family 18 domain (Figure 1B,C). Phylogenetic analysis revealed that *LvChia2* clustered in the same branch as the *Chia2* genes of other crustaceans (Figure 1D). Multiple sequence alignment analysis demonstrated the presence of several highly conserved amino acid sites within the catalytic domain of GH18 (Figure 1E).

### 3.2. LvChia2 Regulates the Metamorphosis of L. vannamei

**Figure 2:**
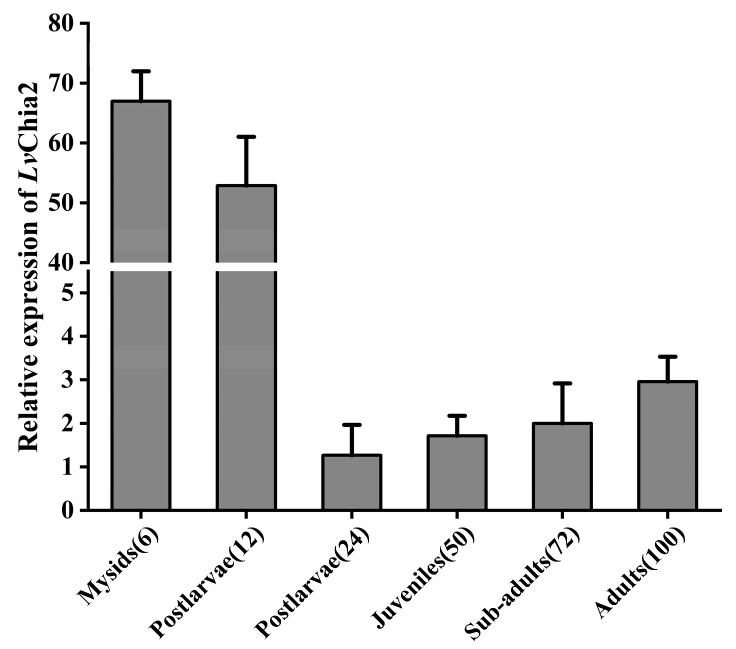
*LvChia2* regulates the metamorphosis of *L. vannamei.* Analysis of *LvChia2* mRNA expression in shrimp at different developmental stages; the age (days post-hatching, DPH) in parentheses is indicated.

The expression level of *LvChia2* in the muscles of shrimp at various developmental stages was assessed. The results indicated that the mRNA expression level of *LvChia2* during the larval stage of shrimp with bran was significantly higher than that in other stages. Although the expression level decreased in the post-larval stage (12 DPH), it remained relatively high. Upon entering the shrimp seedling stage, the expression level was significantly downregulated, suggesting that *LvChia2* may play the role in the metamorphosis of shrimp. During the developmental transition from juvenile to adult shrimp, the expression level exhibited a slight upward trend (Figure 2). These findings suggest that *LvChia2* may have previously unrecognized roles in adult shrimp.

### 3.3. LvChia2 Enhances Lipid Storage Capacity and Promotes Growth in L. vannamei

**Figure 3:**
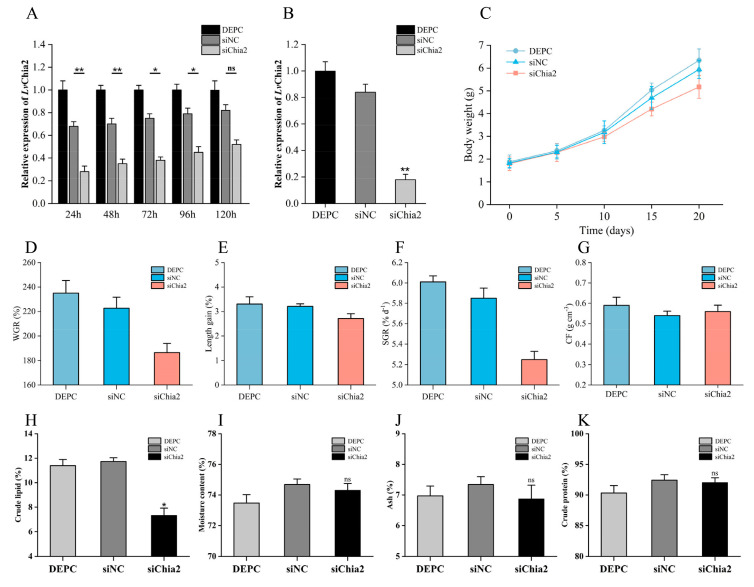
*LvChia2* promotes the growth of *L*. *vannamei* by enhancing its lipid storage capacity. (**A**,**B**) RNA knockdown efficiency of *LvChia2*. The detection of knockdown efficiency was conduct within 120 h (**A**) and the expression level of *LvChia2* after 20 days of knockdown (**B**). (**C**–**G**) Growth performance of shrimp following 20 d of RNAi treatment; changes in body weight (**C**), body length (**D**), weight gain rate (WGR) (**E**), shrimp growth rate (SGR) (**F**) and condition factor (CF) (**G**) after knockdown of *LvChia2*. (**H**–**K**) Analysis of the muscle components of shrimp after knocking down *LvChia2* for 20 d; The changes in crude lipid (**H**) moisture content (**I**), Ash (**J**), and crude protein (**K**) after knockdown of *LvChia2*. The DEPC group was used as the blank control and siNC was used as the negative control. The results are based on three replicate data and shown as mean ± SD (* *p* < 0.05, ** *p* < 0.01, ns *p* > 0.05).

To elucidate the functional mechanism of *LvChia2* in the growth and development of shrimp, RNAi technology was employed to inhibit the expression of *LvChia2*. The knockdown effect (≥50%) persisted for five days following a single siRNA injection (Figure 3A). Consequently, we administered siRNA every five days, and after four rounds of siRNA injections, the knockdown efficiency achieved the desired outcome (>80%) (Figure 3B). In comparison to the two control groups, namely the DEPC- and siNC-treated groups, the weight and body length of the siChia2 group were significantly reduced (Figure 3C,D). Additionally, the weight gain rate and specific growth rate also exhibited significant decreases (Figure 3E,F). However, there were no significant changes in the condition factor, suggesting that the growth inhibition primarily stemmed from the impairment of body shape development rather than alterations in body composition (Figure 3G). Furthermore, no fatalities occurred in any group during the experiment, resulting in a survival rate of 100%. These findings indicate that the knockdown of *LvChia2* significantly inhibited the growth and molting of shrimp without causing mortality. Moreover, following the knockdown of *LvChia2*, the assessment and analysis of muscle nutritional components revealed that, compared to the DEPC control group and the siNC negative control group, the crude lipid content in the siChia2 group was significantly reduced (Figure 3H). However, other nutritional component indicators, including moisture, ash, and crude protein content, did not exhibit statistical differences between the groups (Figure 3I–K). These data suggest that *LvChia2* gene specifically regulates the lipid metabolism process in shrimps. Its functional deficiency primarily impacts the body’s lipid storage capacity, while not significantly interfering with fundamental physiological functions such as protein metabolism and water-salt balance.

### 3.4. Knockdown of LvChia2 Induces Metabolic Reprogramming and Tissue-Specific Oxidative Stress Responses in L. vannamei

**Figure 4:**
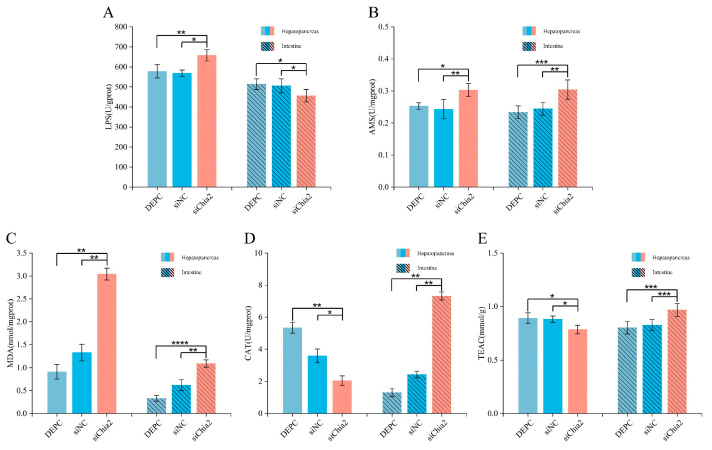
Detection of hepatopancreas and intestinal enzyme activities of *L. vannamei* following knockdown of *LvChia2*. (**A**,**B**) Detection of hydrolase activities (LPS, α-amylase) in the hepatopancreas and intestines of shrimp. (**C**–**E**) Detection of immune-related effects (MDA, CAT, TEAC) in the hepatopancreas and intestines of shrimp. The DEPC group was used as the blank control and siNC as the negative control. The results are based on three replicate data and shown as mean ± SD (* *p* < 0.05, ** *p* < 0.01, *** *p* < 0.001, **** *p* < 0.0001).

Twenty days after the knockdown of *LvChia2*, the LPS activity in the hepatopancreas of the siChia2 group was significantly higher than that in the two control groups, namely the DEPC group and the siNC group, while LPS activity in the intestine was significantly decreased (Figure 4A). This metabolic reprogramming phenomenon indicates that, following the knockdown of *LvChia2*, shrimp compensate for the reduction in lipid storage in muscle tissue by enhancing the lipid decomposition capacity of the hepatopancreas to maintain energy homeostasis. Figure 4B shows that the α-amylase activities in the hepatopancreas and intestines of the siChia2 group were significantly upregulated. This synergistic upregulation pattern contrasts with the alterations in lipid metabolism (increased LPS activity in the hepatopancreas and decreased LPS activity in the intestines), suggesting that the knockdown of *LvChia2* triggered a systematic remodeling of the energy metabolism pattern in shrimp. This adaptive response may help sustain the growth and development of prawns following gene knockdown. Figure 4C indicates that the content of MDA in the hepatopancreas and intestine of the siChia2 group was significantly higher compared to control group, indicating intensified oxidative damage. However, the analysis of antioxidant enzyme activities (Figure 4D,E) revealed that CAT-AOC in the hepatopancreas were significantly decreased. Conversely, CAT activity and T-AOC in the intestinal tract were significantly increased, indicating that the knockdown of *LvChia2* may induce differentiated responses to oxidative stress in shrimp tissues, potentially related to a tissue-specific antioxidant compensation mechanism.

### 3.5. Transcriptome Analysis Following the Knockdown of LvChia2

**Figure 5:**
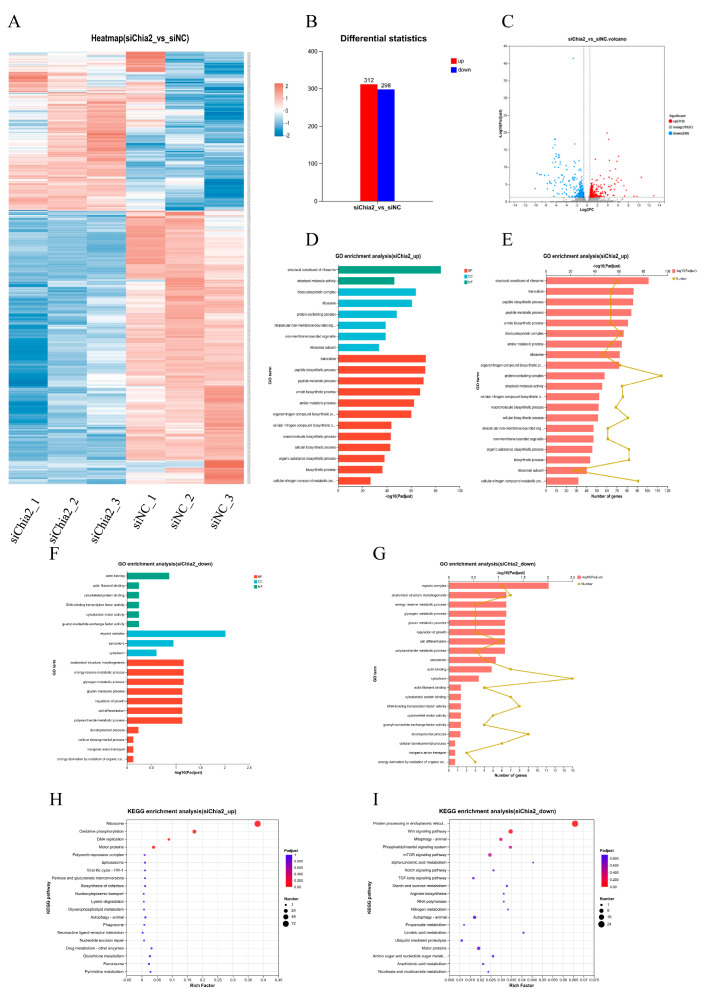
RNA-seq analysis of muscle tissue in *L. vannamei* following *LvChia2* knockdown. (**A**–**C**) RNA-seq analysis of shrimp with *LvChia2* knockdown after 20 d. siNC was injected as a control. (**A**) Cluster plots of DEGs; the horizontal axis represents the sample names and the clustering results of the samples, while the vertical axis represents the DEGs and their clustering results. The colors indicate the expression levels of DEGs in the samples. Histogram (**B**) and volcano plot (**C**) analysis of DEGs in the siChia2 treatment groups. (**D**–**G**) GO classification and enrichment analysis of DEGs in the siChia2 treatment groups. (**D**,**F**) GO classification of DEGs showing increases and decreases; the vertical axis represents the GO terms, and the horizontal axis indicates the significance level of enrichment, corresponding to the height of the columns. A smaller FDR and a larger −log10 (Padjust) value indicate more significant enrichment of the GO term. The three colors represent the three major classifications: biological processes (BP), cellular components (CC), and molecular functions (MF). (**E**,**F**) GO enrichment analysis of DEGs showing increases and decreases; the vertical axis represents the GO terms, while the horizontal axis below indicates the number of genes/transcripts associated with each GO term in comparison, corresponding to different points on the broken line. The horizontal coordinate above represents the significance level of enrichment, corresponding to the height of the bars. A smaller Padjust and a larger −log10 (Padjust) value indicate more significant enrichment of the GO term. (**H**,**I**) KEGG analysis of the top 20 enriched pathways (upregulated pathways in (**H**); downregulated pathways in (**I**)) between siNC and siChia2. The x-axis represents the enrichment factor, while the y-axis represents the pathway names. The color indicates the q-value, and the point size reflects the number of genes. The Rich Factor refers to the value of the enrichment factor.

To clarify the specific mechanism by which *LvChia2* promotes shrimp growth, RNA-seq analysis was conducted on shrimp muscle treated with siNC and siChia2 (Figure 5A). Compared to the siNC-treated group, the siChia2-treated group exhibited a total of 610 DEGs, comprising 312 up-regulated genes and 298 down-regulated genes (Figure 5B,C). GO classification and enrichment analysis revealed that, following the knockdown of *LvChia2*, the DEGs were categorized into (i) Biological Process, (ii) Cellular Component, and (iii) Molecular Function, with all three categories of Molecular Function showing significant enrichment. Among the up-regulated DEGs, a total of 277 GO entries were annotated, primarily involving biological processes such as peptide biosynthesis, amine metabolism, and dynamic regulation of the cytoskeleton (Figure 5D,F). In contrast, the 298 down-regulated DEGs were significantly enriched in 107 GO terms, mainly focusing on glycogen catabolism, regulation of polysaccharide synthesis, and actin binding functions (Figure 5E,G). These results suggest that *LvChia2* plays a crucial role in maintaining homeostasis by coordinating a multi-dimensional regulatory network involving proteins, carbohydrates, and the cytoskeleton.

KEGG enrichment analysis revealed that the knockdown of *LvChia2* primarily impacted Metabolism and Genetic Information Processing, as well as Environmental Information Processing and Cellular Processes (Figure 5H,I). Notably, the upregulated genes were predominantly enriched in metabolic pathways, including amino acid metabolism, energy metabolism, and nucleotide metabolism. Conversely the downregulated genes were significantly associated with genetic information processing pathways, such as the Wnt signaling pathway, the phosphatidylinositol signaling system, and the mTOR signaling pathway. These findings align with the previous results from the GO analysis, collectively indicating that *LvChia2* systematically influences the growth performance of shrimp by integrating a multi-level network of “metabolic reprogramming-gene expression regulation—environmental stress response”, and these results provides a new theoretical framework for studying the molecular mechanisms of growth regulation in crustaceans.

### 3.6. Knockdown of LvChia2 and Its Impact on the Regulation of Core Gene Expression Related to the Growth and Development of L. vannamei

**Figure 6:**
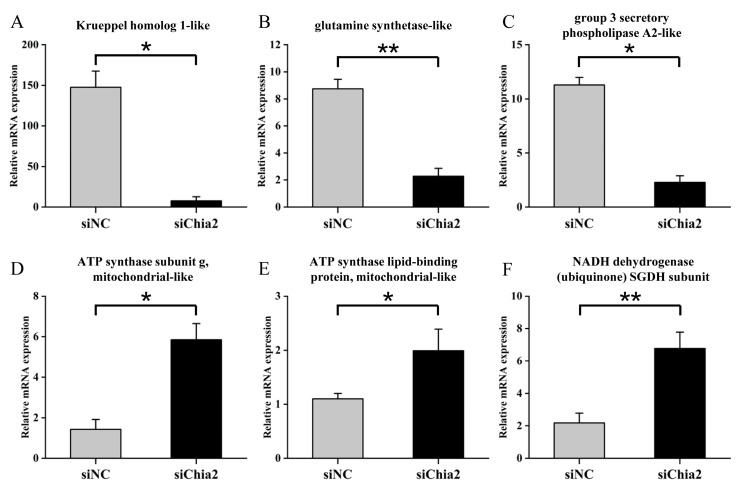
Impact of *LvChia2* knockdown on the regulation of core gene expression related to the growth and development of *L. vannamei*. (**A**–**F**) Correlation analysis between siChia2 and siNC (control) for genes closely associated with growth and development, including *Kr h-1*, *GS*, *sPLA2*, *ATP5L*, *ATP5G*, *ND-SGDH*, using RT-qPCR. The results are based on three replicates and are presented as mean ± SD (* *p* < 0.05, ** *p* < 0.01).

To thoroughly investigate the impact of *LvChia2* knockdown on the growth and development of shrimp, this study identified six core DEGs that are closely associated with these processes from the aforementioned transcriptome through systematic analysis. These genes include ATP synthase subunit (*ATP5L*, GenBank: LOC113809108), ATP synthase lipid-binding protein (*ATP5G*, GenBank: LOC113818783), NADH dehydrogenase subunit (*ND-SGDH*, GenBank: LOC113823157), Krueppel homolog 1 (*Kr-h1*, GenBank: LOC113822590), glutamine synthase (*GS*, GenBank: LOC113823145), and secretory phospholipase A2 (*sPLA2*, GenBank: LOC113820851) (Figure 6A–F). The results indicated that *LvChia2* knockdown led to a significant upregulation of energy metabolism-related genes (ATP synthase and NADH dehydrogenase), while there was a notable downregulation of developmental regulatory genes (Krueppel homolog 1) and nutritional metabolism genes (glutamine synthase and phospholipase A2). These findings suggest that the regulatory effect of *LvChia2* on shrimp growth may be closely linked to the expression regulation of these genes. This implies that *LvChia2* systematically influences the growth and development of shrimp through a three-tiered network mechanism that coordinates energy metabolism reprogramming, molting cycle regulation, and nutrient utilization balance.

## 4. Discussion

In this study, we identified a chitin metabolism-related gene, *LvChia2*, from the transcriptome of *L. vannamei* through weighted gene co-expression network analysis of molting-stage-specific expression profiles, along with validation across different developmental stages. The expression patterns of the chitinase 2 (*Chia2*) gene in various tissues and molting stages suggest that it may play significant roles in the molting process. For example, the *Chia2* homolog in *Eriocheir sinensis* (*EsChia2*) exhibited approximately a 12-fold upregulation in the epidermis and hepatopancreas during the pre-molt stage (D-stage) compared to the intermolt phase (C-phase) [24]. Similarly, the *Chia2* gene in *Portunus trituberculatus* (*PtChia2*) displayed peak expression in the hepatopancreas during the pre-molt stage (C-stage) and in the intestine during the post-molt stage (E-stage), indicating its dual role in immune defense against pathogens and in the degradation of the peritrophic membrane during post-molt remodeling [16]. Consistent with these findings, our study revealed stage-dependent expression dynamics of *LvChia2* in *L. vannamei*, with significantly elevated transcript levels during the mysis larval stage compared to other developmental phases. This expression pattern strongly suggests that *LvChia2* may regulate metamorphic development in shrimp larvae, potentially coordinating cuticle remodeling and developmental transitions through the regulation of chitinolytic activity.

RNA knockdown experiments confirmed that inhibition of *LvChia2* expression resulted in a significant decrease in body weight, body length, and specific growth rate in shrimp, thereby directly verifying its crucial regulatory role in growth performance. Proximate composition analysis revealed that the crude lipid content in the muscle of the knockdown group (siChia2) was significantly reduced, while no significant changes were observed in water content, ash content, or crude protein content. This indicates that the *LvChia2* knockdown primarily affects growth phenotype through targeted regulation of lipid metabolism. In addition, enzyme activity analysis revealed an increase in LPS content in the hepatopancreas and a decrease in LPS content in the intestine. This suggests that shrimp may compensate the reduction in intestinal fat absorption efficiency by activating the lipolysis pathway in the hepatopancreas, such as fatty acid β-oxidation, to break down stored fat, which is consistent with previous research [25]. Furthermore, the analysis indicated that the upregulation of α-amylase in both the hepatopancreas and intestine may partially alleviate lipid metabolism imbalances by enhancing glycolytic energy production, aligning with the “synergistic compensation of metabolic pathways” hypothesis [26]. These findings further confirm the role of *LvChia2* in lipid metabolism. Furthermore, oxidative stress results from an imbalance between the production of reactive oxygen species (ROS) and the antioxidant defense system. Studies have shown that high-fat diets induce lipid peroxidation and hepatopancreatic apoptosis in shrimp, thereby inhibiting their growth and development [27]. The alterations in oxidative stress status caused by *LvChia2* knockdown—such as MDA accumulation in the hepatopancreas and decreased levels of CAT and total antioxidant capacity—suggest that this gene may play a crucial role in maintaining intracellular REDOX homeostasis by regulating the antioxidant system. Conversely, the changes in antioxidant indicators observed in the intestine (including increased CAT and total antioxidant capacity) imply the presence of tissue-specific stress response mechanisms, which may be linked to the interaction of intestinal microbiota or the activation of immune defenses [28]. These findings not only elucidate a novel role for *LvChia2* in the regulation of oxidative stress in crustaceans but also provide critical insights into the interorgan coordination mechanisms that occur during metabolic disturbances in decapods. Understanding these mechanisms will aid in the development of effective antioxidant strategies to enhance productivity and stress resistance in shrimp aquaculture.

Further transcriptome analysis revealed that *LvChia2* influences a wide range of biological processes, including peptide synthesis, glycogen metabolism, cytoskeletal dynamics, and actin binding, by regulating 610 DEGs. Among these, KEGG-enriched metabolic and genetic information processing pathways, such as ATP synthesis and ammonia metabolism, are strongly associated with growth phenotypes. The six core genes identified through screening, including *ATP5L*, *ATP5G*, *ND-SGDH*, *GS*, *sPLA2* and *Kr-h1*, exhibited significant expression differences. Their functional networks suggest that *LvChia2* may regulate growth through several mechanisms. Firstly, energy metabolism regulation plays a crucial role. By upregulating the co-expression of ATP synthase subunits and NADH dehydrogenase, the efficiency of mitochondrial oxidative phosphorylation is enhanced, providing a vital energy foundation for growth. Secondly, ammonia detoxification and nitrogen metabolism are significant processes. By inhibiting the expression of genes like glutamine synthetase, the capacity for ammonia assimilation is limited, resulting in an imbalance in nitrogen metabolism. This imbalance corresponds with the observed phenotype of elevated MDA levels in the hepatopancreas. Finally, cytoskeletal remodeling is another important factor. By downregulating secretory phospholipase A2, membrane phospholipid metabolism is affected, which interferes with cytoskeletal dynamics and subsequently inhibits muscle tissue proliferation. This phenomenon is associated with the observed decrease in weight and body length in the knockdown group. These results closely align with the stress-structural remodeling co-growth model of shrimp [29], further confirming the central role of *LvChia2* in metabolic reprogramming and tissue development.

**Figure 7:**
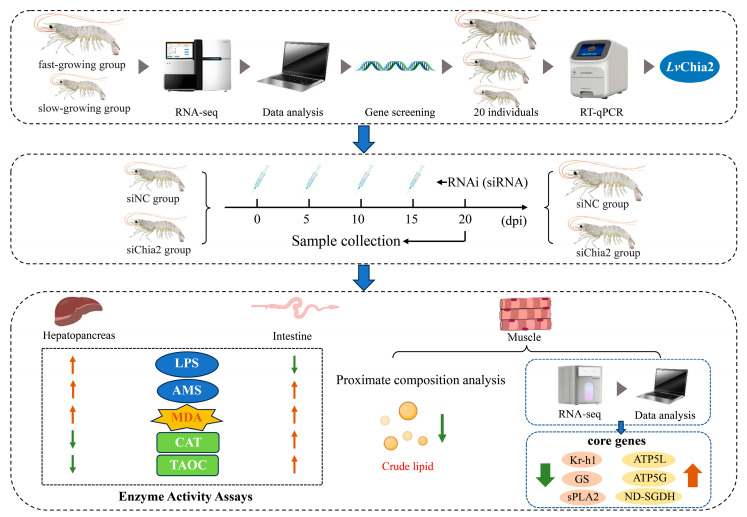
Schematic diagram illustrating the mechanisms by which *LvChia2* affects growth in *L. vannamei*. The arrow pointing upwards indicates an increase, while the one pointing downwards indicates the opposite.

In conclusion, this study elucidates the pivotal role of the *LvChia2* gene in the growth and development of shrimp (*L. vannamei*) by regulating pathways such as lipid metabolism, oxidative balance, and cytoskeletal dynamics. The specific reduction in crude lipid content resulting from its knockdown underscores the significance of this gene in lipid storage regulation. The six key genes (*ATP5L*, *ATP5G*, *ND-SGDH*, *GS*, *sPLA2* and *Kr-h1*) identified in this research provide a novel perspective for analyzing the growth regulatory network of crustaceans. These findings not only enhance our understanding of chitinase function in shrimp but also establish a theoretical foundation for gene-editing approaches in aquaculture (Figure 7).

## Figures and Tables

**Table 1:** Primers used in this study.

Primer	Sequence (5′-3′)	Object
113830447-F	CCTCTGCACGACTCTCATCT	RT-qPCR
113830447-R	AGGCTCGAGACATAGCCATC	RT-qPCR
β-Actin-F	TGAAGATCCTGACGGAGCGT	RT-qPCR
β-Actin-R	GAACCTCTCGTTGCCGATG	RT-qPCR
*LvChia2*-F	GAGCAAGAGGTGGTATGTT	DNA cloning
*LvChia2*-R	CAAAGAAGTAAGCAGGAGTG	DNA cloning
M13-F	CGCCAGGGTTTTCCCAGTCACGAC	PCR screening
MI3-R	AGCGGATAACAATTTCACACAGGA	PCR screening
SiChia2-F	UCUUGUUGACGUCAUACUCCG	RNAi
SiChia2-R	GAGUAUGACGUCAACAAGAGG	RNAi
siNC-F	UUCUCCGAACGUGUCACGUTT	RNAi
siNC-R	ACGUGACACGUUCGGAGAATT	RNAi
113809108-qF	TAGCTCAAGGCCTCGGTAAC	RT-qPCR
113809108-qR	CAAGGGAGCCCTTTCCAATG	RT-qPCR
113818783-qF	ACCACCTCCAAGGACATTGA	RT-qPCR
113818783-qR	AAGAGTTGCTGCTTCAGGGA	RT-qPCR
113823157-qF	GCGCTTCTCGTTTCTCACTT	RT-qPCR
113823157-qR	GTCCAACAGGGATGAGTCCA	RT-qPCR
113822590-qF	TTCACATCCGTACCCACACA	RT-qPCR
113822590-qR	GGCAGTCATTGCAGGAGAAG	RT-qPCR
113823145-qF	AAGACGGTGCTTGACAGGTA	RT-qPCR
113823145-qR	GTCGGCTTCTTGTTGTGCTT	RT-qPCR
113820851-qF	TCTCCTTATGGCGTGCTTCA	RT-qPCR
113820851-qR	TTTCTCCACCTCCTCCTCCT	RT-qPCR

## Data Availability

The datasets supporting this article are included within the article and its Supplementary Information. Other details will be made available on reasonable request.

## Supplementary Materials

The following supporting information can be downloaded at: https://www.mdpi.com/article/10.3390/genes16091110/s1, Figure S1: Correlation between candidate genes expression level and shrimp weight. Key growth-related gene *LvChia2* in *Litopenaeus vannamei* is highlighted by red boxes.

## References

[1] Food and Agriculture Organization of the United Nations (2024). The State of World Fisheries and Aquaculture 2024.

[2] Wang H., Teng M., Liu P., Zhao M., Wang S., Hu J., Bao Z., Zeng Q. (2022). Selection signatures of Pacific white shrimp *Litopenaeus vannamei* revealed by whole-genome resequencing analysis. Front. Mar. Sci..

[3] Vaseeharan B., Rajakamaran P., Jayaseelan D., Vincent A.Y. (2013). Molecular markers and their application in genetic diversity of penaeid shrimp. Aquac. Int..

[4] Argue B.J., Arce S.M., Lotz J.M., Moss S.M. (2002). Selective breeding of Pacific white shrimp (*Litopenaeus vannamei*) for growth and resistance to Taura Syndrome Virus. Aquaculture.

[5] Huang W.J., Leu J.H., Tsau M.T., Chen J.C., Chen L.L. (2011). Differential expression of *Lv*HSP60 in shrimp in response to environmental stress. Fish Shellfish Immunol..

[6] Andriantahina F., Liu X., Feng T., Xiang J. (2013). Current status of genetics and genomics of reared penaeid shrimp: Information relevant to access and benefit sharing. Mar. Biotechnol..

[7] Yu Y., Wang Q., Zhang Q., Luo Z., Wang Y., Zhang X., Huang H., Xiang J., Li F. (2019). Genome scan for genomic regions and genes associated with growth trait in Pacific white shrimp *Litopenaeus vannamei*. Mar. Biotechnol..

[8] Glenn K.L., Grapes L., Suwanasopee T., Harris D.L., Li Y., Wilson K., Rothschild M.F. (2005). SNP analysis of AMY2 and CTSL genes in *Litopenaeus vannamei* and *Penaeus monodon* shrimp. Anim. Genet..

[9] Chen X., Peng M., Yang C., Li Q., Feng P., Zhu W., Zhang Y., Zeng D., Zhao Y. (2024). Genome-wide QTL and eQTL mapping reveal genes associated with growth rate trait of the Pacific white shrimp (*Litopenaeus vannamei*). BMC Genom..

[10] Niu R., Zhang X., Yu Y., Bao Z., Yang J., Yuan J., Li F. (2024). Identification of Growth-Related Gene BAMBI and Analysis of Gene Structure and Function in the Pacific White Shrimp *Litopenaeus vannamei*. Animals.

[11] Liu X., Zhang J., Zhu K.Y. (2019). Chitin in Arthropods: Biosynthesis, Modification, and Metabolism. Adv. Exp. Med. Biol..

[12] Rathore A.S., Gupta R.D. (2015). Chitinases from bacteria to human: Properties, applications, and future perspectives. Enzym. Res..

[13] Fajardo C., De Donato M., Macedo M., Charoonnart P., Saksmerprome V., Yang L., Purton S., Mancera J.M., Costas B. (2024). RNA Interference Applied to Crustacean Aquaculture. Biomolecules.

[14] Yu A., Beck M., Merzendorfer H., Yang Q. (2024). Advances in understanding insect chitin biosynthesis. Insect Biochem. Mol. Biol..

[15] Merzendorfer H., Zimoch L. (2003). Chitin metabolism in insects: Structure, function and regulation of chitin synthases and chitinases. J. Exp. Biol..

[16] Wang W., Wu X.G., Pan G.P., Hou W.J., Cheng Y.X. (2015). Cloning of chitinase and its expression analysis during molting in *Portunus trituberculatus*. J. Fish. China.

[17] Zhang S., Jiang S., Xiong Y., Fu H., Sun S., Qiao H., Zhang W., Jiang F., Jin S., Gong Y. (2014). Six chitinases from oriental river prawn *Macrobrachium nipponense*: cDNA characterization, classification and mRNA expression during post-embryonic development and moulting cycle. Comp. Biochem. Physiol. B Biochem. Mol. Biol..

[18] Zhou K., Jiang S., Huang J., Yang Q., Jiang S., Qiu L., Yang L., Zhou F. (2017). Cloning and expression analysis of Chitinase-2 from *Penaeus monodon* during molting cycle and different larval developmental stages. South China Fish. Sci..

[19] Eichner C., Nilsen F., Grotmol S., Dalvin S. (2014). A method for stable gene knock-down by RNA knockdown in larvae of the salmon louse (*Lepeophtheirus salmonis*). Exp. Parasitol..

[20] Eichner C., Harasimczuk E., Nilsen F., Grotmol S., Dalvin S. (2015). Molecular characterisation and functional analysis of *Ls*Chi2, a chitinase found in the salmon louse (*Lepeophtheirus salmonis*, Krøyer 1838). Exp. Parasitol..

[21] Fajardo C., Martinez-Rodriguez G., Costas B., Mancera J.M., Fernandez-Boo S., Rodulfo H., De Donato M. (2022). Shrimp Immune Response: A Transcriptomic Perspective. Rev. Aquac..

[22] Yang C.L., Chen H.F., Peng M., Li Q.Y., Zeng D.G., Liu Q.Y., Zhao Y.Z., Chen X.H., Chen X.L. (2021). Transcriptome sequencing and screening of genes related to muscle growth and development in *Litopenaeus vannamei*. J. South. Agric..

[23] Kim D., Langmead B., Salzberg S.L. (2015). HISAT: A fast spliced aligner with low memory requirements. Nat. Methods..

[24] Zhang X., Yuan J., Li F., Xiang J. (2021). Chitin synthesis and degradation in crustaceans: A genomic view and application. Mar. Drugs.

[25] Lu J., Tao X., Luo J., Zhu T., Jiao L., Sun P., Zhou Q., Tocher D.R., Jin M. (2023). Dietary choline activates the Ampk/Srebp signaling pathway and decreases lipid levels in Pacific white shrimp (*Litopenaeus vannamei*). Anim. Nutr..

[26] Dissanayake A., Ishimatsu A. (2011). Synergistic effects of elevated CO_2_ and temperature on the metabolic scope and activity in a shallow-water coastal decapod (*Metapenaeus joyneri*; Crustacea: Penaeidae). ICES J. Mar. Sci..

[27] Sun C., Shan F., Liu M., Liu B., Zhou Q., Zheng X., Xu X. (2022). High-fat-diet-induced oxidative stress in giant freshwater prawn (*Macrobrachium rosenbergii*) via NF-κB/NO signal pathway and the amelioration of vitamin E. Antioxidants.

[28] Ning M., Huang Y., Cao X., Shen H., Gu W., Ren X., Meng Q. (2024). The shrimp C-type lectins modulate intestinal microbiota homeostasis in microsporidia infection. Aquaculture.

[29] Zhang Y., Zhuo H., Fu S., Liu J. (2024). Growth performance and growth model fitting of *Litopenaeus vannamei* cultured in pond and factory modes. Aquac. Rep..

