# Supplementary material for: Multidimensional Regulatory Mechanisms of LvChia2 on Growth in the Pacific White Shrimp (Litopenaeus vannamei)
Source: Genes (Basel). 2025 Sep 19;16(9):1110. doi: 10.3390/genes16091110 (PMC12470064; doi:10.3390/genes16091110)
Supplement: Supplementary file 1 [file genes-16-01110-s001.zip › genes-3867278-supplementary.pdf]

Figure. S1

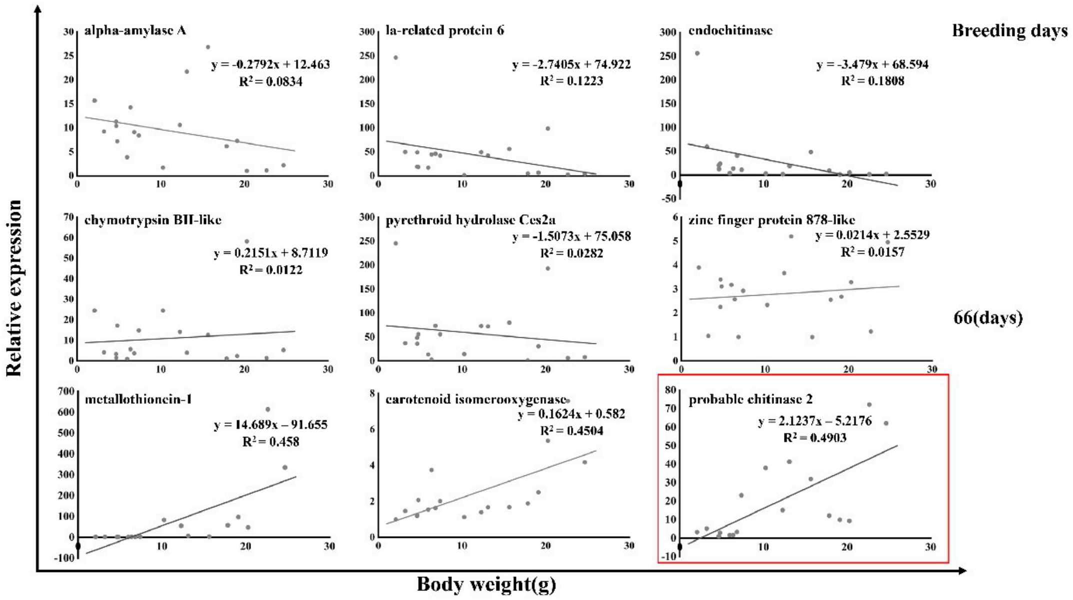

Figure S1. Correlation between candidate genes expression level and shrimp weight. Key growth-related gene *LvChia2* in *Litopenaeus vannamei* is highlighted by red boxes.
